# Comparisons of Vitreal Angiogenic, Inflammatory, Profibrotic Cytokines, and Chemokines Profile between Patients with Epiretinal Membrane and Macular Hole

**DOI:** 10.1155/2021/9947250

**Published:** 2021-07-13

**Authors:** Lu Chen, Weiwei Zhang, Ping Xie, Jiangdong Ji, Huiming Qian, Songtao Yuan, Qinghuai Liu, Zizhong Hu

**Affiliations:** ^1^Department of Ophthalmology, The First Affiliated Hospital of Nanjing Medical University, Nanjing, Jiangsu 210029, China; ^2^Xuzhou Key Laboratory of Ophthalmology, The Municipal Affiliated Hospital of Xuzhou Medical University, Eye Institute of Xuzhou, Xuzhou, Jiangsu 221000, China

## Abstract

**Objectives:**

Idiopathic epiretinal membrane (iERM) or idiopathic macular hole (iMH) is frequently used as a “healthy” control in comparison of vitreous cytokines with other vitreoretinal diseases. This study aimed to investigate if there is a difference in vitreal cytokines expression between patients with iERM and iMH.

**Methods:**

In this prospective study, all subjects received standard pars plana vitrectomy surgery, and 0.5 ml of native vitreous sample was extracted during the vitrectomy. Luminex technology and enzyme-linked immunosorbent assay were used to profile the concentration of 52 classic angiogenic, inflammatory, and profibrotic cytokines and chemokines. Statistical analyses were performed by the Mann–Whitney U test, followed by multiple comparisons by the Bonferroni correction.

**Results:**

Vitreal samples from 13 iERM and 24 iMH were studied. Of the 52 tested cytokines, 41 were similar in expression, and 5 were under the detection limit, while 6 cytokines (MMP-8, Eotaxin, MIP-1a, RANTES, TGF-*β*2, and IL-4) were differently expressed between two groups (*p* < 0.05). Nevertheless, these significances disappeared after the adjustment of Bonferroni correction.

**Conclusion:**

The tested cytokines showed similar expression between iERM and iMH patients. This indicates that eyes with iERM or iMH can be together served as “healthy” controls.

## 1. Introduction

Human vitreous, a complex and transparent extracellular matrix, is composed of approximately 98% water and 2% solid material, including collagen fibrils, hyaluronic acid molecules, and a small amount of proteins and cytokines [1, 2]. Cytokines are important cell-signaling mediators and participate in many conditions [3]. The abnormal expression of vitreal cytokines is acknowledged to be associated with the pathogenesis process, including immunological regulations, inflammation, fibrous hyperplasia, neovascularization, repair processes, and growth mechanisms [4]. As reported, a number of cytokines in vitreous, such as tumor necrosis factor-*α* (TNF-*α*), transforming growth factor-*β* (TGF-*β*), interleukin-1*β*, and so on, are involved in different vitreoretinal diseases [5–7]. To quantify the cytokines in diseased vitreous, most previous studies employed idiopathic epiretinal membrane (iERM) or idiopathic macular hole (iMH) as negative or “healthy” controls because the two diseases are typically localized in the macular zone [5, 7, 8].

ERM may occur idiopathically or secondly to other vitreoretinal diseases, leading to metamorphopsia and vision loss. Retinal glia, retinal pigment epithelial cells, and hyalocytes altogether consist of the fibrocellular tissue [9, 10]. The occurrence of iERM is closely related to posterior vitreous detachment (PVD), which can interrupt the ILM, permitting glial cells, and other cells to migrate and proliferate [9, 10]. The above cellular proliferation and migration are actually promoted by cytokines interaction. Researchers have demonstrated that several cytokines are associated with the pathological membranes in iERM [11]. As for iMH, a full-thickness foveal defect is also responsible for blurred vision, especially central vision deterioration. It is generally accepted that the development of iMH is secondary to abnormal vitreous-retinal traction [12, 13]. Both proliferation and cell migration play a dominant role in iMH development [14].

Despite patients with iMH or iERM were usually selected as “healthy” control, there remains a paucity of evidence on its reasonability and validity. In fact, publications concerning the cytokine levels in the vitreous of patients with iERM and iMH are still rare and controversial. In 2013, Mandal et al. [15] detected that none of the 330 tested proteins differed significantly between the iERM and iMH vitreous groups, while another study [16] conducted in 2016 found that the vitreal levels were significantly higher in iERM than in iMH for 72% of the tested cytokines, indicating that eyes with iERM necessitate a cautious approach to assessing its suitability as a healthy control group [16].

This prospective study was designed to investigate the expression profiles of angiogenic, inflammatory, profibrotic cytokines, and chemokines in the vitreous humor of patients with iERM compared to patients with iMH to evaluate their rationality as a control group. Our results provide a theoretical basis for designing ophthalmology clinical research and may shed light on future etiological studies and therapeutic strategies of iERM and iMH.

## 2. Materials and Methods

### 2.1. Subjects

The study protocol is available at https://clinicaltrials.gov/ (registration date: June 24, 2018; registration ID: NCT03506750). The study adhered to the tenets of the Declaration of Helsinki and was approved by the Ethic Committee of First Affiliated Hospital of Nanjing Medical University (2017-SR-283). Informed written consent was obtained from all patients prior to enrollment.

These patients were treated by standard pars plana vitrectomy surgery under local anesthesia at the First Affiliated Hospital of Nanjing Medical University in the period between March and June 2018. The inclusion criteria were as follows: (1) patients diagnosed with idiopathic ERM and MH (with no concurrent ERM) by optical coherence tomography (OCT) scan (Carl Zeiss Meditec) and (2) patients with iERM or iMH received standard pars plana vitrectomy surgery, and consent was informed. The exclusion criteria were as follows: (1) patients with a history of vitrectomy or other eye surgery; (2) patients with other ocular lesions, such as ocular trauma, uveitis, glaucoma, and diabetic retinopathy; (3) patients with high myopia (<−6D) or pathological myopia; (4) patients with peripheral retinopathy after a thorough examination of the peripheral retinas during surgery, and (5) patients with uncontrolled systematic diseases and underuse of statin [17].

### 2.2. Sample Collection

All native undiluted vitreous samples (approximately 500 *μ*l) were collected with a vitreous cutter at the start of vitrectomy before intraocular infusion. For patients receiving combined phacoemulsfication, vitreous cut and harvest were performed first, followed by compensation of balanced salt solution and phacoemulsfication. The vitreous fluids were centrifuged at 1,500 rpm for 5 min and then immediately frozen at a temperature of −80°C until assay.

### 2.3. Luminex

**Figure 1:**
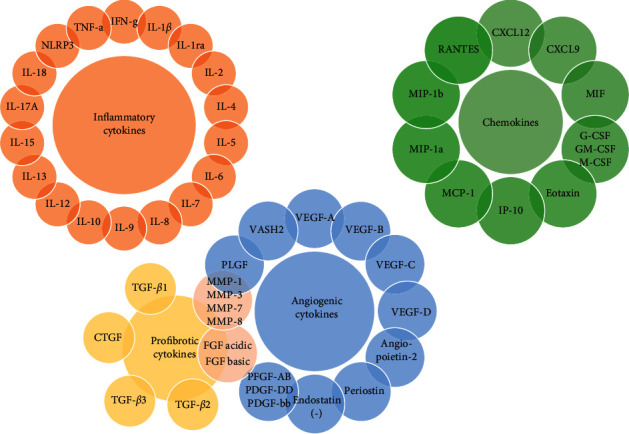
Cytokines measured in this study. The cytokines can be classified into angiogenic (blue), inflammatory (orange), profibrotic cytokines (yellow), and chemokines (green). Such MMP and FGF cytokines were both profibrotic and angiogenic cytokines.

The target cytokines measured in this study were classified into four types: angiogenic, inflammatory, profibrotic cytokines, and chemokines (Figure 1). The concentrations of 48 cytokines were measured with the assistance of Wayen Biotechnologies (Shanghai), Inc., using Luminex multiplex technology (Bio-Rad) according to the manufacturer's protocol. Using this technology, an array of cytokines can be tested and quantified in a single small volume sample. In brief, 50 *μ*l of provided standard or test sample was added to each well of a 96-well microtiter plate. After incubation at room temperature, the samples were incubated with diluted biotin antibody, then with Streptavidin-PE followed by wash buffer. Finally, the plate was read on the Bio-Plex MAGPIX System (Bio-Rad).

### 2.4. Enzyme-Linked Immunosorbent Assay (ELISA)

NLRP3, VASH2, VEGF-B, and CTGF in the vitreous samples were evaluated using Sandwich enzyme-linked immunosorbent assay (ELISA) (MyBioSource ELISA kit, San Diego, CA, and RayBiotech ELISA kit, Norcross, GA) according to each manufacturer's protocol. After color development, the optical density was assayed at 450 nm using a Varioskan flash multifunction plate reader (Thermo Fisher Scientific, Waltham, MA, USA). This experiment was independently repeated three times.

### 2.5. Statistical Analysis

Normally, the concentrations of cytokines below the limit of detection are given a value at 0.5 times the limit of detection for the respective cytokine for data analyses [18, 19]. The statistical software SPSS 22.0 (IBM SPSS Statistics for Macintosh, version 22.0) was used for statistical analyses. Data are reported as mean, standard deviation (SD). First, the normal distribution of data was tested using the Shapiro–Wilk test. Because the assumption of normal distribution was not satisfied, the Mann–Whitney U test was applied for comparisons between groups. *p* values from multiple comparisons were adjusted to 0.001 by the Bonferroni correction.

## 3. Result

### 3.1. Clinical Characteristics of Subjects

**Figure 2:**
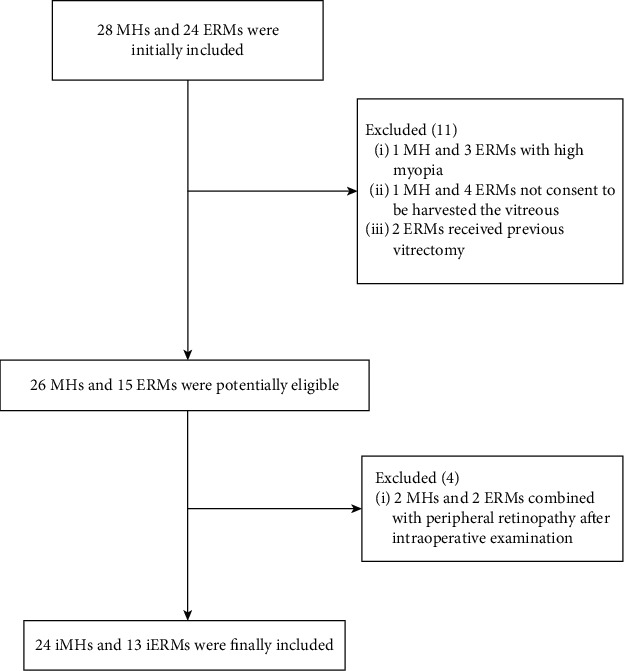
Flow diagram of patients included in this study.

Thirty-eight eyes from thirty-seven patients with iMH or iERM (24 iMH and 13 iERM) were recruited in this study (Figure 2). The clinical characteristics of the subjects are presented in Table 1. The iMH group includes eight males and sixteen females, and the iERM group includes six males and seven females. Though the two groups are majorly composed of females, the distribution of gender does not differ significantly between the two groups (*p*=0.73). The average patient age is similar, and no significant difference is observed in the two groups (iMH: 67.77 ± 8.72 years; iERM: 63.54 ± 6.00 years; *p*=0.1). Furthermore, the mean preoperative vision of iERM group is 0.83 ± 0.25, better than that in the iMH group, 1.14 ± 0.40 (*p*=0.02). The intervals between the first diagnosis by OCT and vitrectomy were 2.07 months in the iMH group and 1.95 months in the iERM group (*p*=0.74). Seven cases (54%) in the iERM group and ten cases (42%) in the iMH group underwent combined cataract surgery (*p*=0.51).

### 3.2. Vitreal Levels of Cytokines

**Figure 3:**
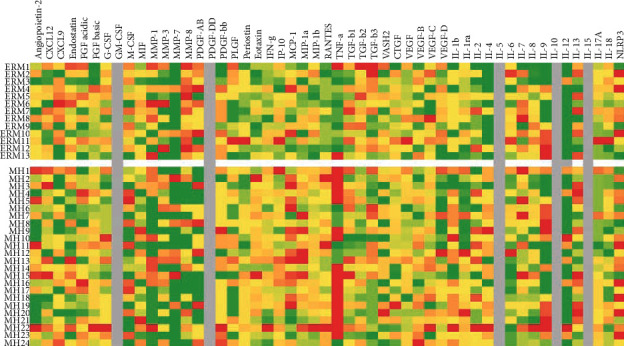
Visualization of the intergroup differences using a heat map chart for all 52 cytokines. Red indicates high expression and green indicates low expression. GM-CSF, PDGF-DD, IL-5, IL-10, and IL-15 in vitreous are too low to be detected (grey).

Using ELISA and Luminex multiplex technology, we determined the profiles of cytokines in the vitreous of subjects. Then the comparison of vitreal cytokine profiles from patients affected by iERMs and iMH was performed. Up to 52 cytokines were measured, and the mean concentrations of these factors in each group were shown in Table 2 and Figure 3.

A statistically significant difference was obtained in the expression of six cytokines between two groups using *p* < 0.05 significance level. These cytokines are TGF-*β*2 (587.05 vs. 458.65 pg/mL; *p*=0.01), IL-4 (0.11 vs. 0.18 pg/mL; *p*=0.002), MMP-8 (342.45 vs. 221.52 pg/mL' *p*=0.002), eotaxin (4.12 vs. 5.14 pg/mL; *p*=0.03), MIP-1a (0.25 vs. 0.29 pg/mL; *p*=0.04), and RANTES (1.3 vs. 1.71 pg/mL; *p*=0.002). However, these differences were not significant after the Bonferroni correction (*p* < 0.001 as statistically significant).

Thirteen of the fifty-two tested cytokine levels were less than the lowest detected concentration of the standard, and thus, the multiparameter standard curve was used to calculate the concentration of these cytokines (Figure 3). However, the concentrations levels of five of them lay below the linear range of the standard curve, which means that the content of GM-CSF, PDGF-DD, IL-5, IL-10, and IL-15 in vitreous of patients with iERM and iMH is too low to be detected.

## 4. Discussion

Idiopathic epiretinal membrane and idiopathic macular hole are two of the most common vitreomacular interface diseases. Many studies, involving the measurement of intraocular cytokines, usually employed iERM and iMH as controls for retinal pathologies such as proliferative diabetic retinopathy, uveitis, retinal vein occlusion, and high myopia [5, 7, 8, 20]. However, the comparative data of the vitreal cytokine levels in patients with ERMs and MHs are very sparse. In the present study, we found no difference in the vitreal cytokine profiles between Asian patients with iERM and those with iMH. According to our study results, the vitreous fluid in the eyes of patients with either iERM or iMH can be served as controls in further research.

As iERM is one of the vitreous macular traction diseases, its pathophysiology mechanism remains unclear. A generally received opinion is that age-related vitreoretinal interface changes and pathologic cell proliferation results in vertical or horizontal traction force to the macula [21, 22]. A number of cellular factors are involved in cell recognition and signal transduction during the occurrence and development of iERM formation, among which TGF-*β*2 is the most extensively studied factor [6, 21, 23]. The concentration of vitreal TGF-*β*2 level we measured is 587.05 ± 88.89 pg/mL, while already published data are 327.98 ± 99.58 pg/mL and 951.06 ± 593.25 pg/mL [6]. The discrepancy between these data could be due to differences in the tested methods and test kits. Of note, in Figure 3, the heat map shows the overall expression of all 52 cytokines where the red indicates the highest and the green indicates the lowest expression. TNF-*α* was measured using Luminex multiplex technology, and the expression was relatively low both in ERM and MH, ranging from 0.94 pg/mL (green) to 2.94 pg/mL (red). Though the TNF-*α* seems higher in most cases in MH compared with those in ERM, the Mann–Whitney U test detected no difference with statistical significance. Future studies might be needed to further verify the difference with a larger sample size or more sensitive method, such as ultrasensitive single-molecule array (Simoa) [24].

Our data suggested that before the Bonferroni correction, the TGF-*β*2 levels in the vitreous fluids of the patients with iERM were significantly higher than those in iMH (*p*=0.01). These findings were similar to those reported by Ludovico Iannetti, whose research found that TGF-*β*2 and nerve growth factor (NGF) were associated with idiopathic ERMs [6]. However, in our research, the statistical difference disappeared after the Bonferroni correction. In addition to iERM or iMH, Iannetti et al. once used primary retinal detachment (within 72 hours of retinal detachment onset) as a control group [6]. Ideally then, the human vitreous of healthy subjects should be used as the control. However, in clinical practice, a vitreous sample from healthy subjects cannot be obtained because of the ethical issue. The present study demonstrates that the cytokine expression profile between patients with iERM and those with iMH is similar, which means that like iMH, iERM can be reliably used as control groups.

Before our CONCEPT trial, we thoroughly retrieved the published literature on vitreous cytokines during the last decade. Considering the compatibility of different cytokines in a single commercial bead array, we finally investigated 48 cytokines using the multiplex bead array method and the other 4 cytokines with ELISA. Here, a total of 52 cytokines were included in the present study, covering most of the published factors associated with intraocular diseases and some new cytokines in other related diseases, such as asthma and Alzheimer's disease. And these cytokines can be classified into four main groups: the inflammatory factors and chemokines, the promoting angiogenesis factors, and the fibrogenic cytokines, which corresponding, respectively, to inflammatory, neovascular, and fibrotic retinopathy. Among those cytokines, the frequently reported cytokines such as TNF-*α* [25], IL-6 [7, 26], and TGF-*β* [27] were compared between our study and proliferative diabetic retinopathy, and the expressions of the three cytokines in proliferative diabetic retinopathy were found times higher as compared with those in MH/ERM.

There were published studies focusing on the protein or cytokine profiles of vitreous from patients with iERM and iMH but yielding opposite conclusions [15, 16, 28]. Nakul Mandal et al. demonstrated that none of the 330 protein spots changed significantly between the iERM and iMH groups using comparative proteomic experiments. On the contrary, Souska Zandi et al. employed ELISA and multiplex technology, while Zhang et al. employed one-dimensional gel fractionation and liquid chromatography–tandem mass spectrometry analyses, and both detected some differently expressed cytokines between ERM and MH. However, several issues should be kept in mind to interpret the controversial findings between ours and others. First, the expression of cytokines may be duration-related. The average course of iERM in our study was 1.95 months, while the duration of iERM in Souska Zandi's study was unknown. Among the included patients, those with vision loss more than 6 months constituted 94% of the ERM group in the Souska Zandi's study and 62% in our study. Besides, in 31.9% of the measured patients in Souska Zandi's study, the cytokine concentrations lay below the lower cutoff level. Though half of the lower cutoff value was used for subsequent calculation, the result would be still in error. Third, the race might also be involved in the difference of cytokine levels in the vitreous. The above-mentioned studies were conducted in Switzerland, Europe, and the USA, while our patients were all Asian populations.

We acknowledge that this study has several limitations. First, the number of patients included is limited, and the clinic data might be uneven in the two groups. Further study is warranted as the variables such as hypertension, diabetes mellitus, and cataract also need to be still evaluated in a larger sample size to confirm the results. This work will be more of clinical significance since it defines the inclusion/exclusion criteria for the iERM/iMH group to be used as a control group in future studies. Second, we did not compare the vitreous cytokines among the two macular diseases with vitreous floaters. However, the safety and efficiency of vitrectomy over Nd:YAG laser for floaters remain to be demonstrated. Thus, most studies involving the cytokines measurements still employed iERM or iMH as “negative” controls. [5, 7, 8, 20]. Third, we did not include ERM or MH eyes with high myopia. ERM and MH are two of the main complications of pathological myopia. It will be interesting to investigate the vitreous cytokines between emmetropic and myopic eyes in conditions of ERM or MH. Fourth, not comparing the vitreous levels of cytokines with other vitreoretinal diseases such as diabetic retinopathy and proliferative vitreoretinopathy is also a limitation for our study.

## 5. Conclusions

In conclusion, the current research provides substantial evidence that the cytokine in the vitreous shows similar expression in iERM and iMH groups. This prospective control study is the first one that demonstrates Asian patients with iERM and iMH that can be chosen as “negative” control. These results provide a theoretical foundation for future clinical design methods. Further investigations with more participants should be conducted to verify these findings.

## Figures and Tables

**Table 1:** Clinic characteristics of patients with iMH and iERM.

	iMH	iERM	*p* value
Number	24	13	—
Gender (m/f)	8/16	5/8	0.73
Age (years)	67.77 ± 8.72	63.54 ± 6.00	0.1
Smoking status	7	5	0.56
Hypertension	10	5	0.85
Diabetes	4	2	0.58
Vision (LogMAR)	1.14 ± 0.40	0.83 ± 0.25	0.02
Axial length	23.9 ± 0.99	23.9 ± 0.81	0.95
Duration (months)	16.3	13.2	0.72
Combined cataract surgery	14	6	0.51

*Note.* m: male; f: female; and data are expressed as mean ± SD.

**Table 2:** Comparisons of 52 vitreal cytokines between iERMs and iMHs.

Cytokine	iERM			iMH			*p* value
	Mean (pg/mL)	SD	Se	Mean (pg/mL)	SD	Se	
Angiopoietin-2	93.17	49.34	17.44	123.87	116.90	33.75	0.97
CXCL12	539.87	126.12	44.59	582.49	157.83	45.56	0.53
CXCL9	135.57	94.06	42.06	114.76	51.79	14.95	0.96
Endostatin	62,975.80	12,018.00	4,249.01	57,561.62	17,190.33	4,962.42	0.45
FGF acidic	36.96	3.98	1.41	37.45	4.15	1.20	0.80
FGF basic	4.41	2.27	0.63	5.83	1.19	0.24	0.08
G-CSF	3.73	0.28	0.10	3.79	0.43	0.12	0.91

*GM-CSF* ^#^							
M-CSF	154.86	62.47	22.08	141.57	98.68	28.49	0.74
MIF	7,995.97	3,911.00	1,382.75	11,903.57	12,912.18	3,727.43	0.85
MMP-1	50.97	18.08	6.39	41.24	21.25	6.13	0.43
MMP-3	190.62	178.07	62.96	160.49	112.80	32.56	0.65
MMP-7	196.21	69.45	24.56	281.02	215.86	62.31	0.57
MMP-8	342.45	42.58	15.05	221.51	93.32	26.94	0.00*∗*
PDGF-AB	41.87	17.61	6.23	27.66	17.71	5.11	0.07

*PDGF-DD* ^#^							
PDGF-bb	5.63	2.12	0.75	6.66	2.14	0.62	0.52
PLGF	2.97	2.84	0.79	2.34	1.53	0.31	0.91
Periostin	2,615.99	4,663.18	2,085.44	582.38	344.35	99.41	0.65
Eotaxin	4.12	1.05	0.37	5.14	1.94	0.56	0.03*∗*
IFN-g	43.06	9.75	2.70	51.32	24.47	5.00	0.20
IP-10	884.75	1,071.70	297.24	1,032.97	920.38	187.87	0.34
MCP-1	136.27	27.54	9.74	149.56	46.91	13.54	0.48
MIP-1a	0.25	0.05	0.02	0.29	0.05	0.01	0.04*∗*
MIP-1b	1.70	0.74	0.20	2.04	1.25	0.36	0.68
RANTES	1.30	0.19	0.07	1.71	0.35	0.10	0.00*∗*
TNF-a	1.54	0.51	0.18	1.65	0.51	0.15	0.68
TGF-*β*1	363.34	155.10	43.02	362.57	155.24	31.69	0.98
TGF-*β*2	587.05	88.89	24.65	458.65	117.30	23.94	0.01*∗*
TGF-*β*3	1.91	0.70	0.19	1.60	0.57	0.12	0.15
VASH2	0.08	0.01	0.01	0.08	0.02	0.01	0.77
CTGF	13.30	4.43	1.23	12.90	6.17	1.32	0.60
VEGF-A	24.06	21.43	9.59	17.69	6.99	2.02	0.36
VEGF-B	0.04	0.02	0.01	1.00	2.68	0.95	0.82
VEGF-C	446.85	290.81	80.66	488.22	202.90	41.42	0.62
VEGF-D	113.07	61.02	16.92	101.15	85.30	17.48	0.28
IL-1*β*	0.07	0.01	0.00	0.10	0.05	0.01	0.06
IL-1ra	10.14	5.33	1.48	17.07	20.70	4.22	0.91
IL-2	0.19	0.10	0.04	0.26	0.09	0.03	0.15
IL-4	0.11	0.02	0.01	0.18	0.07	0.02	0.00*∗*

*IL-5* ^#^							
IL-6	13.72	22.66	10.13	9.64	17.93	5.18	0.65
IL-7	16.13	6.36	2.25	15.48	5.43	1.57	0.68
IL-8	113.07	61.02	16.92	101.15	85.63	17.48	0.28
IL-9	0.08	0.10	0.06	0.27	0.24	0.07	0.23

*IL-10* ^#^							
IL-12	0.16	0.00	0.00	0.23	0.14	0.04	0.38
IL-13	0.48	0.38	0.14	0.48	0.33	0.11	1.00

*IL-15* ^#^							
IL-17A	0.89	0.70	0.19	0.78	0.39	0.08	0.89
IL-18	69.45	35.09	9.73	57.16	15.62	3.19	0.31
NLRP3	5.35	2.03	1.01	4.72	1.97	0.74	0.65

^#^The cytokine concentrations lay below the limits of detection; ^*∗*^*p* < 0.05; however, these differences were not significant after Bonferroni correction (*p* < 0.001 as statistically significant).

## Data Availability

The data used to support the findings of this study are available from the corresponding author upon request.

## Abbreviations

TNF-*α*: Tumor necrosis factor-*α*
TGF-*β*: Transforming growth factor-*β*
IL: Interleukin
iERM: Idiopathic epiretinal membrane
iMH: Idiopathic macular hole
ILM: Inner limiting membrane
PVD: Posterior vitreous detachment
ELISA: Enzyme-linked immunosorbent assay
GM-CSF: Granulocyte-macrophage colony-stimulating factor
PDGF: Platelet-derived growth factor.
